# Role of Mitochondria-Associated Endoplasmic Reticulum Membrane in Inflammation-Mediated Metabolic Diseases

**DOI:** 10.1155/2016/1851420

**Published:** 2016-12-15

**Authors:** Themis Thoudam, Jae-Han Jeon, Chae-Myeong Ha, In-Kyu Lee

**Affiliations:** ^1^Department of Biomedical Science, Graduate School, Kyungpook National University, Daegu, Republic of Korea; ^2^BK21 Plus KNU Biomedical Convergence Program, Kyungpook National University, Daegu, Republic of Korea; ^3^Leading-edge Research Center for Drug Discovery and Development for Diabetes and Metabolic Disease, Kyungpook National University, Daegu, Republic of Korea; ^4^Department of Internal Medicine, School of Medicine, Kyungpook National University, Daegu, Republic of Korea

## Abstract

Inflammation is considered to be one of the most critical factors involved in the development of complex metabolic diseases such as type 2 diabetes, cancer, and cardiovascular disease. A few decades ago, the discovery of mitochondria-associated endoplasmic reticulum (ER) membrane (MAM) was followed by the identification of its roles in regulating cellular homeostatic processes, ranging from cellular bioenergetics to apoptosis. MAM provides an excellent platform for numerous signaling pathways; among them, inflammatory signaling pathways associated with MAM play a critical role in cellular defense during pathogenic infections and metabolic disorders. However, induction of MAM causes deleterious effects by amplifying mitochondrial reactive oxygen species generation through increased calcium transfer from the ER to mitochondria, thereby causing mitochondrial damage and release of mitochondrial components into the cytosol as damage-associated molecular patterns (DAMPs). These mitochondrial DAMPs rapidly activate MAM-resident inflammasome components and other inflammatory factors, which promote inflammasome complex formation and release of proinflammatory cytokines in pathological conditions. Long-term stimulation of the inflammasome instigates chronic inflammation, leading to the pathogenesis of metabolic diseases. In this review, we summarize the current understanding of MAM and its association with inflammation-mediated metabolic diseases.

## 1. Introduction

Overnutrition leads to metabolic and cellular derangement, which contributes to chronic low-grade inflammation, termed “metainflammation” [1]. Inflammation plays a crucial role in the development of metabolic disorders, which are implicated in the progression of many diseases, including diabetes, cardiovascular disease, and cancer [2]. Drastic changes in cellular metabolism resulting from excessive nutrient intake negatively affect the function of the endoplasmic reticulum (ER) and mitochondria [3–5]. Mitochondria and the ER have their own distinct roles in regulating cellular homeostasis, but these organelles also physically interact, exchanging calcium ions, lipids, and other metabolites to maintain cellular bioenergetics and integrity [6]. The contact region between the ER and mitochondria is known as mitochondria-associated ER membrane (MAM). Recently, intense study of the ER and mitochondrial dynamics has revealed a number of mechanisms involved in the development of inflammation and insulin resistance [6, 7]. The interaction between the ER and mitochondria is an important cellular process, which occurs rapidly to maintain normal cellular function [8, 9]. However, abnormal induction of this ER-mitochondrial association has been observed in various cell types and tissues under pathological conditions [6, 10–13].

MAM was first identified by J. E. Vance and D. E. Vance a few decades ago, when they described this interface as a major site for the synthesis and transfer of phospholipids [14]. In addition, calcium transfer from the ER to mitochondria via MAM regulates cellular bioenergetics by activating calcium-sensitive dehydrogenase, phosphatase, and adenosine triphosphate (ATP) synthase to drive mitochondrial respiration and ATP synthesis [15–18]. Calcium overloading in mitochondria and calcium depletion in the ER due to prolonged induction of MAM lead to mitochondrial and ER dysfunction, thereby resulting in activation of inflammatory and cell death signaling pathways [13, 19–21]. MAM provides a platform for various proteins that control numerous cellular pathways. Of these, inflammasome components and other inflammatory factors play a critical role in initiating inflammatory responses at the MAM interface [7, 22].

In this review, we describe the role of MAM in initiating inflammation and its role in the development of inflammatory diseases.

## 2. Cellular Organelle-Mediated Inflammation

### 2.1. ER Stress-Associated Inflammation

ER is a cellular organelle that is specialized for protein synthesis, protein folding, and membrane lipid synthesis [38]. The ER lumen can also store a large amount of calcium, which is utilized by ER chaperones for protein folding and to maintain cellular calcium homeostasis [39, 40]. Under conditions of metabolic stress, such as hypoglycemia, hyperglycemia, or elevated fatty acid levels, ER stress is activated in many tissues [41, 42]. Recent reports show that metabolic diseases such as diabetes, obesity, atherosclerosis, and cancer are linked with aberrant ER stress and inflammation [42]. There are three major independent pathways that control ER stress, namely, the pancreatic ER kinase (PERK), inositol-requiring enzyme 1 (IRE-1), and activating transcription factor 6 (ATF6) pathways, which collectively comprise the unfolded protein response (UPR). All these UPR pathways are responsible for inducing the transcription of genes encoding proinflammatory cytokines [43, 44].

The IRE-1 alpha branch is a well-established UPR pathway. Under normal conditions, IRE-1 is bound to the ER chaperone protein glucose-regulated protein 78 (GRP78), but, under conditions of stress, IRE-1 is liberated from GRP78 and undergoes oligomerization, and its ribonuclease domain becomes activated by autophosphorylation. Activated IRE-1 catalyzes the splicing of X-box-binding protein 1 (XBP-1) mRNA [45] and promotes XBP-1-mediated induction of tumor necrosis factor alpha (TNF-*α*) and interferon beta (IFN-*β*) gene expression [46]. In addition, IRE-1 promotes interleukin-1 beta (IL-1*β*) gene expression, via activation of glycogen synthase kinase 3 beta (GSK-3*β*), but the mechanism of this effect is less clear [47].

ER stress also results in PERK-dependent activation of the Janus kinase 1/signal transducer and activator of transcription (JAK-1/STAT-3) signaling pathway, which induces expression of the inflammatory cytokine interleukin-6 (IL-6) and that of several chemokines, including C-C motif ligand 2 (CCL2), C-C motif ligand 11 (CCL11), and C-C motif ligand 20 (CCL20), in neuronal cells, which drive neuroinflammation [48]. Moreover, CCAAT/enhancer-binding protein homologous protein (CHOP), downstream of PERK, promotes expression of interleukin-23 (IL-23), probably by binding to a specific recognition sequence in its promoter region, which has been identified in dendritic cells [42, 49]. IL-23 is a key mediator of inflammation, because it stabilizes T-helper 17 cells [50].

**Figure 1:**
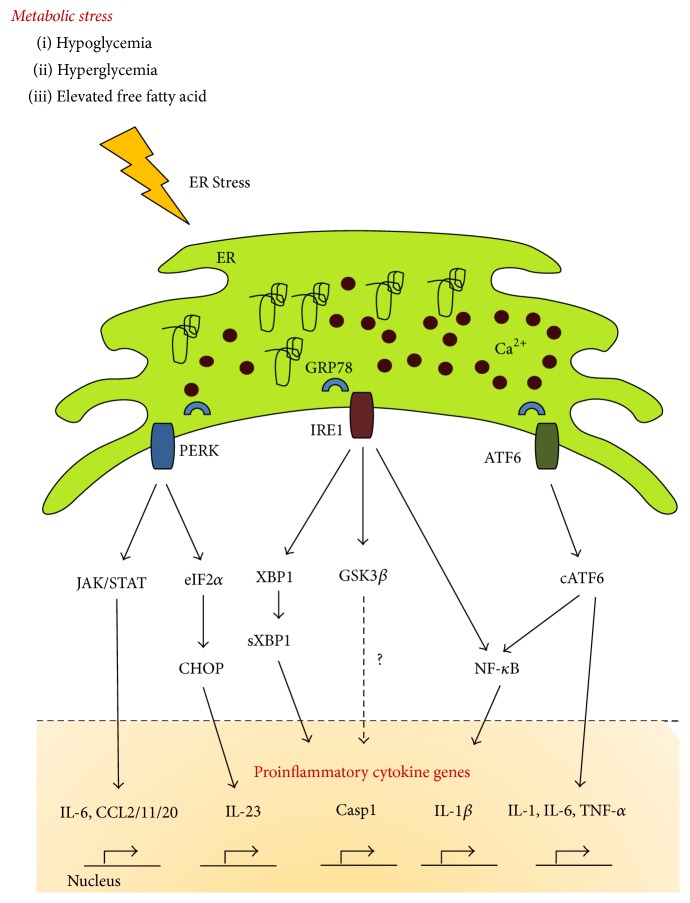
Metabolic stress-mediated induction of an inflammatory response via ER stress signaling pathways. Metabolic stressors such as hypoglycemia, hyperglycemia, and elevated free fatty acids induce endoplasmic reticulum (ER) stress pathways that increase production of inflammatory cytokines. PERK activation increases IL-6, IL-23, CCL2, CCL11, and CCL20 gene expression via the JAK/STAT and eIF2*α*-CHOP signaling pathways. IRE1 activation results in increased secretion of a diverse group of proinflammatory cytokines through XBP1, GSK3*β*, and the NF-*κ*B signaling cascade. Lastly, ER stress promotes ATF6 cleavage in the Golgi complex, which triggers proinflammatory cytokine production via the NF-*κ*B pathway. GRP78: glucose-regulated protein 78; PERK: pancreatic ER kinase; IRE1: inositol-requiring enzyme 1; eIF2*α*: eukaryotic initiation factor 2 alpha; CHOP: CCAAT-enhancer-binding protein homologous protein; JAK: Janus kinase; STAT: signal transducer and activator of transcription; XBP1: X-box-binding protein 1; sXBP1: spliced X-box-binding protein 1; ATF6: activating transcription factor 6; cATF6: cleaved activating transcription factor 6; GSK*β*: glycogen synthase kinase 3 beta; NF-*κ*B: nuclear factor-kappa B; IL-6: interleukin-6; IL-23: interleukin-23; TNF-*α*: tumor necrosis factor alpha; IL-1*β*: interleukin-1 beta; CCL2: chemokine (C-C motif) ligand 2; CCL11: chemokine (C-C motif) ligand 11; CCL20: chemokine (C-C motif) ligand 20; Casp1: caspase-1.

Lastly, ATF6 is an ER protein that is cleaved into its active form in the Golgi complex during ER stress, which is followed by its translocation into the nucleus, where it functions as a transcription factor to induce acute phase response (APR) genes. The APR commences during the early phases of the innate immune response, involving increased expression of proinflammatory factors, including IL-1, IL-6, and TNF-*α*. ER stress-induced ATF6 expression enhances nuclear factor-kappa B (NF-*κ*B) signaling, which in turn inhibits the anti-inflammatory protein kinase B (PKB/AKT) signaling pathway, thereby enhancing the inflammatory immune response in a mouse model of liver ischemia-reperfusion (IR) injury [51]. An overview of the ER stress-associated inflammatory signaling pathways and their downstream products is shown in Figure 1.

### 2.2. Mitochondrial Stress-Associated Inflammation

Mitochondria represent cellular power plants, synthesizing ATP through oxidative phosphorylation. They are composed of outer and inner membranes, delineating the intermembrane space, and matrix. They are dynamic cellular organelles that regulate calcium signaling, apoptosis, metabolism, and inflammatory responses [52]. Moreover, they are the primary source of reactive oxygen species (ROS), which are derived from the respiratory chain and drive proinflammatory signaling, NF-*κ*B expression, and cytokine production [53–55]. Mitochondria are also an important site for NACHT, LRR, and PYD domain-containing protein 3 (NLRP3) inflammasome activation [7]. NLRP3 detects damaged mitochondria and initiates inflammatory responses [56]. As a consequence of NLRP3 inflammasome formation, activated caspase-1 promotes release of proinflammatory cytokines, including IL-1*β* and IL-18 [57]. In response to viral infection, accumulated and aggregated mitochondrial antiviral-signaling proteins (MAVS) on the mitochondrial outer membrane (OMM) activate interferon regulatory factor 3 (IRF3) and NF-*κ*B [58]. The specific mechanism of this effect will be discussed later in this review.

Mitochondrial dysfunction plays a major role in type 2 diabetes (T2D) [59]. Systemic dysfunction of mitochondria leads to inflammation in pancreatic *β*-cells and impaired regulation of blood glucose [57]. This relationship between inflammation and pancreatic cell dysfunction was demonstrated by the use of an IL-1 receptor antagonist to ameliorate hyperglycemia in T2D [60]. Mitochondrial DNA (mtDNA) mutation resulting from oxidative stress is also an etiological factor in the development of rheumatoid arthritis (RA). mtDNA mutation is associated with the induction of proinflammatory cytokines, such as TNF-*α*, and IFN*γ* [61]. Furthermore, as part of its proinflammatory effect, TNF-*α* promotes ROS production [61]. Mitochondrial dysfunction increases inflammatory responses in normal human synoviocytes, and these are ameliorated by antioxidants, including mitoTEMPO, N-acetylcysteine, and resveratrol [62]. Mitochondria also have multiple roles in cardiac pathophysiology. Sterile inflammation (in the absence of microorganisms) in the heart involves mitochondrial dysfunction [63]. For example, damage-associated molecular patterns (DAMPs) generated from mtDNA induce systemic inflammatory responses during pressure overload [64]. Taken together, these findings support the critical role of mitochondrial dysfunction in chronic inflammatory diseases.

## 3. Role of Mitochondria-Associated ER Membrane

### 3.1. Role of MAM in Lipid Metabolism

MAM is enriched with enzymes involved in phospholipid synthesis [65, 66]. Phospholipids are synthesized at the ER-mitochondrial interface, because each organelle does not possess all the required enzymes to complete the whole process [67]. Phosphatidylethanolamine (PE) and phosphatidylcholine (PC) are among the most abundant phospholipids on the ER membrane. The precursor phosphatidic acid (PA) is converted into phosphatidylserine (PS) by phosphatidylserine synthases 1 and 2 (PSS1 and PSS2) in the ER. PS is then transferred to mitochondria, where phosphatidylserine decarboxylase (PSD) converts it to PE. Lastly, PE is transferred to the ER from mitochondria to synthesize PC using phosphatidylethanolamine methyltransferase 2 (PEMT2) [68]. PC is a preferred component of lipid droplet packaging and lipoproteins [69]. The PC/PE ratio is maintained within a narrow range, and an inappropriate ratio has been identified as a major factor in the progression of steatohepatitis [70]. An increase in the PC/PE ratio was also observed in obese mice, which affected ER calcium-restoring capacity by inhibiting activity of the ER calcium importer sarco/endoplasmic reticulum Ca^2+^-ATPase (SERCA) [5].

MAM is also actively involved in cholesterol transport into mitochondria and steroidogenesis in steroid-synthesizing cells, for example, mouse Leydig cells and rat adrenal gland. Cholesterol transport within mitochondria and steroidogenic activity is dependent upon the expression level and stability of steroidogenic acute regulatory protein (StAR), which is abundant at the MAM. StAR interacts with another MAM-resident protein, voltage-dependent anion channel 2 (VDAC2), and this interaction was found to be crucial for StAR translocation at the MAM, before it is targeted to mitochondria for its role in steroidogenesis [71].

### 3.2. Role of MAM in Calcium Homeostasis

Another pivotal role of MAM is in calcium transport. The OMM ion channel voltage-dependent anion channel (VDAC), the chaperone protein glucose-regulated protein 75 (GRP75), and the ER calcium channel inositol 1,4,5-triphosphate receptor (IP3R) form a macromolecular complex to mobilize calcium from the ER to mitochondria. Knocking down of GRP75 disrupts this complex and reduces mitochondrial calcium uptake [72]. In addition, calcium exchange between the ER and mitochondria can occur through another ER calcium channel, ryanodine receptor (RyR), and VDAC [37, 73]. Apposition of mitochondria and ER via MAM provides a sturdy platform to maintain a strong calcium concentration gradient around the mitochondrial calcium channel VDAC, allowing it to take up calcium more efficiently [74, 75]. Following this, the mitochondrial calcium uniporter (MCU), located in the mitochondrial inner membrane, imports calcium from the mitochondrial intermembrane space to the mitochondrial matrix [76]. At a physiological concentration of matrix calcium, aerobic metabolism and ATP synthesis are stimulated by activation of calcium-sensitive mitochondrial metabolic enzymes [77]. During the extreme rise in matrix calcium that can occur in pathological conditions, enhanced ROS generation occurs, which eventually leads to a collapse in mitochondrial function and apoptosis, via opening of mitochondrial permeability transition pores (mPTPs) [17, 78, 79]. Taken together, it is likely that accumulation of mitochondrial calcium, occurring because of an abnormal induction of MAM, leads to apoptosis via mPTP opening.

### 3.3. MAM as an Interorganelle Platform for Cellular Signaling Pathways

MAM harbors numerous proteins that play a crucial role in cellular metabolism, growth, and survival. AKT kinase is well known for its role in cell proliferation. Hyperactive AKT signaling pathways are frequently observed in many human cancers [80]. In vitro and in vivo studies have found that AKT inhibits ER calcium channel IP3R activity via direct phosphorylation, thereby limiting calcium release from the ER [81, 82]. ER calcium release via IP3R has been reported to play a key role in initiating intrinsic apoptosis by modulating mitochondrial calcium accumulation [81]. The C-terminal tail of all three isoforms of the inositol 1,4,5-trisphosphate (IP3) receptor, namely, IP3R1, IP3R2, and IP3R3, carries a conserved consensus RXRXX (S/T) substrate motif for AKT kinase [82]. Mutations in the AKT phosphorylation site of IP3R promote ER calcium release mediated by cellular stress and apoptosis. Overexpression of constitutively active AKT dramatically reduces ER calcium release, decreases mitochondrial calcium levels, and prevents apoptosis initiated by various calcium-dependent apoptotic stimuli [83]. These findings shed light on the mechanism that allows cancer cells to escape cell death pathways activated by the hyperactivation of AKT signaling pathways. Interestingly, AKT translocates at the MAM interface, which allows it to regulate IP3R activity and MAM integrity [19, 28]. Growth hormone-regulated mTOR Complex 2 (mTORC2), which plays a role in cellular proliferation, resides at the ER membrane and MAM, where it induces calcium mobilization from the ER to mitochondria. mTORC2 activates AKT by phosphorylation, thereby inhibiting IP3R3 and suppressing calcium release from the ER to mitochondria. In addition, mTORC2-mediated AKT activation potentiates MAM integrity by phosphorylating phosphofurin acidic cluster sorting protein 2 (PACS2), an ER protein that tethers mitochondria at the MAM interface [19].

Phosphatase and tensin homolog (PTEN) is a tyrosine phosphatase that preferentially dephosphorylates phosphoinositide substrates and negatively regulates insulin action by counteracting the effects of PI3-kinase, which converts phosphatidylinositol (3,4,5)-bisphosphate (PIP2) to phosphatidylinositol (3,4,5)-trisphosphate (PIP3) [84]. Furthermore, suppression of PTEN protects against insulin resistance in diet-induced obese (DIO) and* ob*/*ob* mice models [85, 86]. PTEN is located in a number of subcellular compartments, including the plasma membrane, cytoplasm, mitochondria, and nucleus [87–89]. Interestingly, PTEN is also enriched in MAM, where it can regulate ER to mitochondrial calcium mobilization [28]. In addition, PTEN reverses the AKT-mediated IP3R3 phosphorylation that terminates this calcium flux. The increased calcium influx from the ER to mitochondria mediated by MAM-localized PTEN induces mitochondrial calcium-dependent apoptosis as a tumor suppressing mechanism [28].

GSK-3*β* is an enzyme that phosphorylates and inactivates glycogen synthase [90, 91]. It protects cells from death by manipulating the threshold for mPTPs under conditions of cardiac hypertrophy and IR injury [92, 93]. Surprisingly, a recent study revealed that GSK-3*β* is relocated to MAM, where it regulates IP3R1-mediated calcium release, thereby inducing calcium accumulation in mitochondria and mPTP pore opening. Inhibition of GSK-3*β* could thus play a critical role in cardioprotection during IR injury [24].

## 4. MAM Association with Cellular Stress Pathways Which Triggers Inflammation

### 4.1. Hypoxia

Reduction in oxygen availability during hypoxia alters cellular metabolism; oxidative phosphorylation in mitochondria is decreased, and, instead, cellular ATP generation mainly relies on glycolysis [94]. Hypoxia induces synthesis of the transcription factor hypoxia-inducible factor 1*α* (Hif1*α*). Hif1*α* promotes glucose catabolism by inducing transcription of several enzymes involved in glycolysis [95, 96]. Moreover, it suppresses pyruvate metabolism in mitochondria by inducing pyruvate dehydrogenase kinase 1 (PDK1) transcription. Hypoxia and inflammation are very closely related mechanisms in metabolic diseases [97, 98]. Individuals with acute mountain sickness and healthy hikers who are exposed to hypoxia at high altitude have higher levels of circulating proinflammatory cytokines [99, 100]. Moreover, hypoxia is a major factor causing inflammatory diseases such as atherosclerosis, RA, inflammatory bowel disease, and colorectal cancer [101–103]. In obese subjects, hypoxia is observed in rapidly enlarged adipose tissue depots. Here, inflammation is also observed, evidenced by increased infiltration with macrophages [104]. Enlarged adipose tissue depots secrete inflammatory cytokines such as monocyte chemoattractant protein-1 (MCP-1), IL-6, IL-1, and TNF-*α*, which can initiate inflammatory reactions [105, 106]. Conversely, the anti-inflammatory adipokine adiponectin, which plays a protective role in diabetes and atherosclerosis [107], was found to be reduced in obese subjects [108, 109] and adipocytes exposed to hypoxia [110–113].

Interestingly, FUN14 domain-containing 1 (FUNDC1), a protein present in the mitochondrial membrane, was previously found to mediate hypoxia-induced mitophagy by directly interacting with the autophagosome component microtubule associated protein 1 light chain 3 (LC3) protein in mammalian cells [114]. Recently, it was also found to interact with the ER resident protein calreticulin at the MAM interface and to act as an adaptor for dynamin-related protein 1 (Drp1) recruitment in mitochondria, thereby inducing hypoxia-mediated mitochondrial fission and mitophagy [115]. This report therefore revealed a close relationship between hypoxia and MAM. Further study is required to understand the importance of MAM in mediating inflammatory responses under hypoxic conditions.

### 4.2. Mitochondrial Dynamics

#### 4.2.1. Mitochondrial Fusion

Mitochondria actively undergo fusion and fission processes to maintain their functions. Mitochondrial fusion is considered to be crucial for cellular survival and recovery after stressful conditions [116]. This process is accomplished by the action of mitofusin 1 (Mfn1) and mitofusin 2 (Mfn2), present in the OMM, and optic atrophy 1 (OPA-1), present in the mitochondrial inner membrane. Mitochondria that are unable to regain their function undergo fission to promote mitophagy, or, in the case of severe mitochondrial damage, undergo apoptosis [117]. Mfn1 and Mfn2 are crucial proteins modulating mitochondrial tethering, docking, and fusion [118]. During mitochondrial fusion, Mfn1 helps tethering adjacent mitochondria in a GTP-dependent manner, whereas Mfn2 is tethered with low efficiency. Mfn1 and Mfn2 form homotypic or heterotypic complexes during mitochondrial fusion in the mammalian cell [119, 120]. In addition, Mfn2 is proposed to be a prominent candidate tethering ER and mitochondria by localizing to the MAM interface. In addition to its localization to the mitochondrial outer membrane, Mfn2 also localizes to the ER membrane. Its localization to the ER membrane allows it to bind to Mfn1 or Mfn2 present on adjacent mitochondria, which promotes the formation of a bridge between the ER and mitochondria. Subsequently, Mfn2 deficiency leads to a reduction in mitochondrial calcium uptake rate [121].

However, several conflicting results questioning the indispensability of Mfn2 as an ER-mitochondria tether have been reported recently [122–124]. One of the reports showed that cells lacking Mfn2 or cells treated with siMfn2 still maintain contact between the ER and mitochondria. This finding challenges numerous other reports describing Mfn2 as a prominent marker for MAM formation [123]. However, we cannot rule out the possibility that other MAM tethers maintain integrity and compensate for the absence of Mfn2. Surprisingly, another independent study showed that Mfn2-deficient cells manage to maintain close proximity between the ER and mitochondria; however, these cells are less sensitive to MAM mediated mitochondrial calcium uptake than control cells and do not show disruption in the mitochondrial calcium transport system. This finding supports the notion that Mfn2 is still an essential component [125]. Mitofusins have been reported to be involved in several metabolic diseases [117, 126]. The role of Mfn-1 has been much less explored than that of Mfn-2. A previous study found that cardiomyocyte specific Mfn1 knockout (KO) mice exhibit normal left-ventricular function and that mitochondrial respiration is normal in cardiomyocytes isolated from the Mfn-1 KO heart. Surprisingly, Mfn1 KO cardiomyocytes are less sensitive to mitochondrial depolarization and show higher cell viability than wild-type cardiomyocytes after challenge with hydrogen peroxide [127]. Another report showed that Mfn1 overexpression leads to hyper fusion of mitochondria, which negatively affects mitochondrial function and mitochondrial motility in INS-1E rat clonal beta cells [128]. On the other hand, the role of Mfn2 has been extensively studied by numerous groups because it is associated with many metabolic syndromes. Charcot-Marie-Tooth disease type 2A has been linked with an Mfn2 mutation [129] that results in the loss of Mfn2 activity [130]. Moreover, Mfn2 expression levels are reduced in skeletal muscle of obese and diabetic humans [131]. In addition, rats fed with HFD for 8 weeks have lower Mfn2 gene expression than control rats, and this lower expression is accompanied by attenuated insulin signaling in the liver. Overexpression of Mfn2 compensates for HFD-mediated disruption of insulin signaling [132]. Liver-specific Mfn2 KO mice have higher levels of mitochondrial fragmentation and glucose intolerance and lower responses to insulin in the liver. Deficiency of Mfn2 also negatively affects mitochondria function by reducing mitochondrial respiration and enhancing ROS generation in the mouse liver [133]. Interestingly, an Mfn2 loss-of-function mutant shows impaired glucose, pyruvate, and palmitate oxidation caused by low OXPHOS complex subunits expression, whereas a Mfn2 gain-of-function mutant shows higher mitochondrial metabolism in skeletal muscle cells caused by high OXPHOS complex subunit expression [134]. Cardiomyocytes lacking Mfn2 are more viable against ischemia-reperfusion heart injury by reducing mitochondrial calcium overload and ROS generation and by delaying mPTP pore opening [135].

#### 4.2.2. Mitochondrial Fission

Mitochondrial fission involves the Drp1 protein, which is recruited from the cytosol to the OMM by various adaptors such as mitochondrial fission protein 1 (Fis-1), mitochondrial fission factor (MFF), mitochondrial dynamics protein of 49 kDa (MiD49), and mitochondrial dynamics protein of 51 kDa (MiD51), which are present on the OMM [114]. In this context, MAM plays an important role during the fission process by wrapping the damaged mitochondria with ER membrane, thereby promoting Drp1 translocation to the ER-mitochondria interface, where it can cleave mitochondria efficiently and target damaged mitochondria for mitophagy [136–138]. The connection between inflammation and mitochondrial fission is well documented: the bacterial component lipopolysaccharide (LPS) induces Drp1 translocation from the cytosol to mitochondria and promotes mitochondrial fission, accompanied by increased expression of genes encoding proinflammatory cytokines. By contrast, blocking Drp1 translocation to mitochondria or knocking down the Drp1 gene results in downregulation of proinflammatory cytokine gene expression in LPS-stimulated microglial cells [139].

### 4.3. Autophagy

Autophagy is an essential cellular mechanism that is required for degrading damaged cellular components and removing them from cells, to protect them from further damage. Extensive research in the field of autophagy has revealed that autophagosome formation originates in the MAM [140].

Autophagy plays an important role during bacterial and viral infections; the specific term for the degradation of foreign pathogens by autophagy is “xenophagy.” Studies of genetically and diet-induced obese mouse models demonstrated a suppression of autophagy in liver, adipose tissue, and beta cells [141–143]. Moreover, DIO mice displayed impaired autophagy in macrophages, provoking polarization into the proinflammatory M1 subtype [144]. This suppression of autophagy increased the accumulation of damaged intracellular organelles and amplified the inflammatory response [64, 145–147]. In addition, induction of autophagy in macrophages protected against bacterial infection and suppressed damaging inflammation [148–150]. However, direct evidence of the importance of MAM in linking autophagy and inflammation is still lacking, and therefore this would be an interesting area for future studies.

## 5. Inflammatory Signaling at the MAM Interface

### 5.1. NLRP3

NLRP3, also known as cryopyrin, is a member of the nucleotide-binding domain and leucine-rich repeat-containing (NLR) protein family. NLR proteins are involved in the induction of inflammatory responses in response to invading foreign pathogens and intracellular, cellular, or tissue damage. NLR proteins recognize diverse pathogenic molecules, when they become activated and assemble into inflammasome complexes with other inflammasome components. These complexes then promote proinflammatory cytokine secretion as an immunologic response [151]. Unlike other NLR family members, NLRP3 recognizes not only foreign pathogenic particles but also DAMPs released from damaged mitochondria [152].

NLRP3 is expressed in most tissues but predominantly in macrophages. It localizes to the ER membrane in its resting state and relocates to MAM in its activated state, where it detects increased ROS production from damaged mitochondria. It is worth noting that increased ER-mitochondrial association induces calcium accumulation in mitochondria and exacerbates mitochondrial dysfunction and ROS production. Moreover, calcium accumulation is the major factor that causes opening of mPTP and thereby the release of mitochondrial components that activate the inflammasome via NLRP3. Furthermore, increased NLRP3 inflammasome activation and IL-1*β* secretion are observed after inhibition of mitophagy/autophagy by 3-methyladenine (3MA) treatment or after knocking down the autophagy regulator beclin 1 and autophagy protein 5 (ATG5) in macrophages, resulting in accumulation of damaged mitochondria and increased ROS generation. Similarly, induction of mitochondrial damage using a series of mitochondrial respiratory chain inhibitors amplifies IL-1*β* secretion. However, treatment with the antioxidant 4-amino-2,4-pyrrolidinedicarboxylic acid (APDC) blocked NLRP3 inflammasome activation and IL-1*β* secretion [7].

Small heterodimer partner (SHP) negatively regulates NLRP3 inflammasome activation by inhibiting binding of NLRP3 to apoptosis-associated speck-like protein containing a CARD (ASC) protein. Hence, LPS stimulation in SHP-deficient macrophages leads to accumulation of damaged mitochondria and sustained interaction between NLRP3 and ASC protein in the ER, which is accompanied by increased secretion of IL-1*β* and IL-18. Excessive IL-1*β* and IL-18 secretion is typically observed in kidney tubular necrosis with peritoneal gout, diabetes, atherosclerosis, and Alzheimer's disease (AD) [153]. In addition, the ER-initiated UPR activates the NLRP3 inflammasome to contribute to ER stress-mediated chronic inflammation, while blockade of NLRP3-induced IL-1*β* release or blockade of the IL-1 receptor improves glucose homeostasis [154, 155].

### 5.2. ASC

ASC is an important component of the inflammasome [156], and it forms a complex with NLRP3 to recruit caspase-1 and promote IL-1*β* maturation and secretion. ASC proteins are predominantly localized to the cytosol, but a small proportion are also found in the ER under unstimulated conditions. Stimulation with the microbial toxin nigericin or monosodium urate crystals increased ASC translocation at MAM and also to a lesser extent in mitochondria. Its translocation at MAM was found to be a NLRP3-dependent mechanism. ASC was found to be crucial for IL-1*β* maturation and secretion, because ASC KO cells significantly reduced IL-1*β* secretion [7]. Furthermore, ASC knockout mice are protected from high fat diet-induced hepatic insulin resistance, hepatic steatosis, and adipocyte hypertrophy [157, 158]. Proinflammatory cytokines such as IL-1*β* and IL-18 are considered to be important factors linking inflammation to insulin resistance [159, 160]. Caspase-1-deficient mice are also protected from diet-induced obesity. IL-1*β* production and macrophage infiltration in adipose tissue were significantly lower in these mice [157]. In summary, therefore, MAM provides an ideal platform for the activation and assembly of the NLRP3, ASC, and caspase-1 inflammasome complex, which is required for the maturation of the proinflammatory cytokines, IL-1*β* and IL-18.

### 5.3. MAVS

MAVS is a protein that has been previously known as virus-induced signaling adaptor (VISA), IPS-1, and cardif. It is localized in peroxisomes and mitochondria and plays a major role during hepatitis C virus (HCV) infection. HCV invasion is recognized by retinoic acid-inducible gene-1 protein (RIG-I), a pattern recognition receptor (PRR) that activates MAVS to initiate an antiviral response through NF-*κ*B and interferon regulatory factor 1 (IRF-1) signaling pathways [161–163]. Interestingly, MAVS was found to localize at the MAM in human hepatoma cells (HUH7 cells) [161, 164]. During HCV infection, cytosolic RIG-I detects the dsRNA of HCV, triggering its relocation to the MAM to activate MAVS. As a result, activated MAVS plays a crucial role in the activation of NF-*κ*B and IFN-*α* production. However, during chronic HCV infection, the NS3/4A protease produced by HCV is translocated to the MAM to specifically cleave MAVS at this location, thereby suppressing the MAVS-mediated proinflammatory response [161]. In addition, MAVS detects viral RNA through its interaction with RIG-I and melanoma differentiation-associated protein 5 (MDA-5), resulting in increased expression of IFN-*β* [165]. Most importantly, MAVS is as important as ASC for inflammasome activation, because MAVS acts as an adaptor for NLRP3, and is required for its optimal function and recruitment to mitochondria, where IL-1*β* but not IFN-*β* is generated (Figure 3). Therefore, MAVS plays a crucial role in inflammasome formation, thereby promoting IL-1*β* production in response to tissue injury and necrosis [166].

### 5.4. *α*-Synuclein

The neurodegenerative process that occurs during Parkinson's disease (PD) is caused by inflammation in neuronal cells [167]. Previous reports have identified increased levels of TNF-*α* and IL-1*β* in the striatum and in peripheral blood mononuclear cells in patients with idiopathic PD [168–171]. These findings have also been corroborated by many other groups using PD mouse models [172–174]. *α*-Synuclein is an abundant protein in brain, which, when mutated, causes toxic gain of function and abnormal aggregation of Lewy bodies, leading to the development of PD [175]. Moreover, this abnormal aggregation activates microglial cells and increases the production of proinflammatory cytokines [167].

Synuclein is predominantly localized in the cytosol [176] and to a lesser extent in mitochondria [177, 178], but its presence in MAM suggests its involvement in the development of PD. Interestingly, pathological mutation of *α*-synuclein leads to disruption of ER-mitochondrial interaction and is accompanied by reduced MAM activity. Surprisingly, suppression of MAM in cells carrying mutant *α*-synuclein resulted in increased mitochondrial fragmentation, which was independent of the fission inducer Drp1. Consistent with this, reexpression of wild-type *α*-synuclein in cells carrying the mutant version rescued mitochondria from fragmentation. Taken together, wild-type *α*-synuclein plays a crucial role in maintaining mitochondrial health, but its mutation, which is normally observed in PD, causes mitochondrial fragmentation, which is consistent with the increased number of damaged mitochondria that are present in PD [34]. However, it was reported that PD patients with a parkin RBR E3 ubiquitin protein ligase (PARK2) mutation showed enhanced MAM formation, an observation that was replicated using PARK2 KO mice [33], and which implicates an alternative mechanism for the development of PD.

### 5.5. Presenilins

Presenilin protein is well known for its association with AD [179, 180]. It is a component of *γ*-secretase enzymes, which are responsible for cleaving amyloid precursor protein (APP) into amyloid beta (A*β*). Presenilin mutations lead to increased aggregation and accumulation of A*β*, which is the key pathogenic factor in AD [181, 182]. Two isoenzymes exist, presenilin-1 (PS1) and presenilin-2 (PS2), and both proteins were found to be enriched in MAM fractions from neuronal and nonneuronal cells. Presenilin found in MAM is functional; thus APP cleavage into A*β* can occur on MAM [183]. Interestingly, MAM formation was found to be upregulated in PS mutant cells and in fibroblasts from both familial and sporadic cases of PD [10]. However, PS2 requires interaction with mitofusin 2 to positively modulate calcium exchange between the ER and mitochondria. In addition, AD-linked PS2 mutants are more effective than wild-type PS2 in tethering the ER and mitochondria [32].

Inflammation is actively involved in the pathogenesis of AD [184]. AD that is linked with a PS mutation is characterized by increased levels of MCP-1, IL-6, and IL-8 release, while a PS1 mutation in microglial cells amplified TNF-*α*, IL-1*α*, IL-1*β*, and IL-6 gene expression [185]. All these findings highlight the importance of the MAM-localized protein PS in the progression of AD.

## 6. Stress Sensors at the MAM Interface

### 6.1. PERK

**Figure 2:**
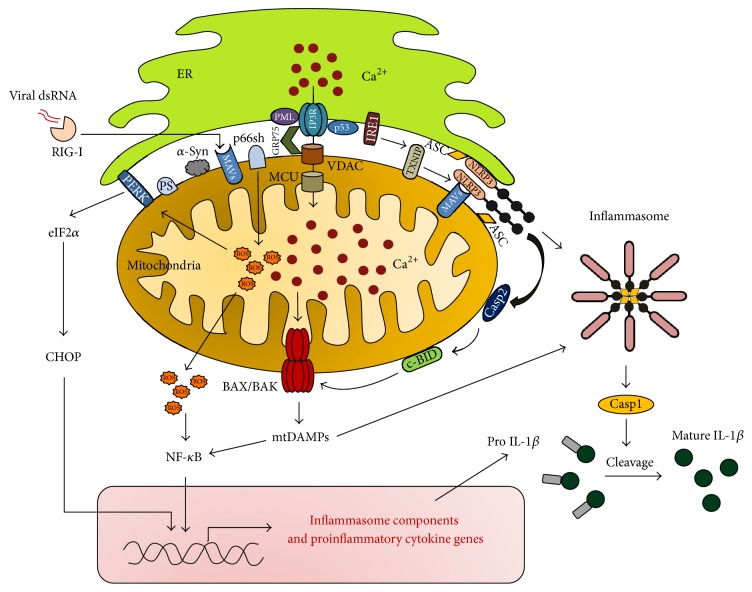
Mitochondria-associated ER membrane as an inflammatory signaling hub. Close contact between the endoplasmic reticulum (ER) and mitochondria via mitochondria-associated ER membrane (MAM) formation allows the ER calcium channel IP3R to funnel calcium into mitochondria by forming a complex with the chaperone protein GRP75 and the mitochondrial outer membrane calcium channel VDAC. Then, the inner mitochondrial membrane calcium channel MCU injects calcium into the mitochondrial matrix. Under pathological conditions, elevated ER-mitochondrial interaction promotes calcium accumulation via proteins present at the MAM interface. The MAM-resident proteins PML and p53 control ER calcium release by physically interacting with IP3R. In addition, p66sh amplifies mitochondrial ROS production. Increased cellular ROS activates NF-*κ*B, a master regulator of inflammation, resulting in increased expression of genes encoding numerous proinflammatory cytokines. PERK plays a dual role by tethering ER and mitochondria and detecting ROS production, which triggers its ER stress signaling cascade. In response to ER stress, activated IRE1 promotes NLRP3 through a TXNIP-dependent mechanism. NLRP3 detects mitochondrial ROS generation and recruits caspase-2 (Casp2) to mitochondria to cleave BID, forming c-BID, which in turn promotes BAX/BAK-mediated mtDAMP release. NLRP3 activation also promotes recruitment of ASC to MAM, where inflammasome complexes are assembled. Finally, inflammasomes activate caspase-1 (Casp1), which cleaves pro-IL-1*β* to generate mature IL-1*β*. Foreign pathogen-associated dsDNA is detected by RIG-1, resulting in activation of MAVS and therefore promotion of its downstream inflammatory response. MAVS can also recruit NLRP3 onto mitochondria and promote inflammasome formation, which specifically induces IL-1*β* production. Other MAM-resident proteins such as presenilins (PS) and *α*-synuclein (*α*-Syn) play a crucial role in the pathogenesis of Alzheimer's disease and Parkinson's disease, respectively. IP3R: inositol 1,4,5-trisphosphate receptor; MCU: mitochondrial calcium uniporter; GRP75: 75 kDa glucose-regulated protein; VDAC: voltage-dependent anion channel; PERK: pancreatic ER kinase; PML: promyelocytic leukemia protein; p53: tumor protein p53; p66sh: 66 kDa isoform of the growth factor adapter Shc; NF-kB: nuclear factor-kappa B; eIF2*α*: eukaryotic initiation factor 2 alpha; CHOP: CCAAT-enhancer-binding protein homologous protein; IRE1: inositol-requiring enzyme 1; NLRP3: NACHT, LRR, and PYD domain-containing protein 3; ASC: apoptosis-associated speck-like protein containing a CARD; TXNIP: thioredoxin-interacting protein; IL-1*β*: interleukin-1 beta; BID: BH3 interacting-domain death agonist; ROS: reactive oxygen species; BAX: Bcl-2-associated X protein; BAK: Bcl-2 homologous antagonist/killer; MAVS: mitochondrial antiviral-signaling protein; RIG-1: retinoic acid-inducible gene-1 protein; mtDAMPs: mitochondrial damage-associated molecular patterns.

Under normal conditions, PERK is bound to the GRP78 ER chaperone protein, but, during ER stress, GRP78 is released from PERK and activated by autophosphorylation. Its activation suppresses translation by phosphorylating eukaryotic initiation factor 2 alpha (eIF2*α*) at serine 51 and reduces transcription of I kappa B alpha (I*κ*B*α*), which leads to hyperactivation of NF-*κ*B and increased inflammatory cytokine production [186, 187]. PERK is an ER membrane protein that detects increases in mitochondrial ROS and activates the UPR signaling pathway. PERK is found abundantly in MAM, where its role is not confined to the activation of its canonical pathway that is eIF2*α*-ATF4-mediated induction of the proapoptotic factor CHOP but also encompasses ROS detection and ER and mitochondrial tethering (Figure 2) [188]. PERK-mediated CHOP induction increases the transcription of BAX protein, which inhibits the antiapoptotic OMM protein B-cell lymphoma 2 (Bcl-2) and participates in mPTP pore opening.

### 6.2. PML

Promyelocytic leukemia protein (PML) is a tumor suppressor protein that mediates multiple apoptotic responses [189]. PML localization in MAM promotes calcium-dependent apoptotic cell death. Under normal conditions, PML forms a protein complex with IP3R3, AKT, and protein phosphatase 2 (PP2a), which regulates cell fate by increasing mitochondrial calcium and apoptosis, but, in cancer cells, the expression of PML is generally low, making them resistant to cell death [189].

### 6.3. p53

Tumor protein p53 (p53) activity regulates cell cycle arrest and apoptosis in malfunctioning cells. It is a transcription factor that regulates expression of DNA repair proteins and initiates the production of apoptotic signals when it detects damaged and irreparable DNA [190, 191]. p53 has been found in many subcellular compartments, including the nucleus, cytosol, and mitochondria, and its proapoptotic effects when localized to mitochondria are transcription-independent [192]. However, p53 accumulation in the ER and MAM was recently reported in cancer cells treated with an anticancer drug, which promoted mitochondrial calcium accumulation, followed by mitochondrial fragmentation and apoptosis [26].

### 6.4. p66Shc

The MAM-resident protein 66 kDa isoform of the growth factor adapter Shc (p66Shc) regulates mammalian cell life span by increasing ROS generation (Figure 2). Previously, it was found to reside within mitochondria, by virtue of a mitochondrial targeting sequence [193], but its exact location within mitochondria is still controversial [194, 195]. Its activity is highly increased in aged mice due to increased phosphorylation by protein kinase C beta (PKC*β*) in the MAM fraction, which results in increased ROS generation and cellular senescence [27].

## 7. Role of MAM in Mitochondrial DAMP Generation

The evolutionary origin of mitochondria from a bacterial endosymbiont suggests that components released from damaged mitochondria may act as a trigger for mechanisms used to detect damaged cells and drive immunologic responses [196].

Two common factors that initiate inflammation are pathogen-associated molecular patterns (PAMPs) and DAMPs [196]. Components of microorganisms such as LPS, endotoxins, and flagellin, found on bacterial cell membranes, are considered to be the prototypical class of PAMPs. PAMPs are recognized by toll-like receptors (TLRs) and PRRs present on the host cell membrane [197]. By contrast, DAMPs are generated within host cells in response to cellular damage caused by extensive cellular stress, either in the absence or in the presence of pathogenic infection [198]. DAMP-triggered inflammation in the absence of any foreign pathogen is defined as sterile inflammation, and it has been widely studied due to its critical role in the development of inflammatory diseases [199, 200]. Mitochondria-derived DAMPs such as ATP, ROS, mtDNA, cytochrome C, cardiolipin, succinate, and N-formyl peptide play a pivotal role in the activation of NLRP3-mediated inflammatory responses [201].

Another recent study revealed that* Brucella abortus* strain RB51 infection in mouse bone marrow-derived macrophages (BMDMs) leads to induction of mitochondrial DAMPs. RB51 infection induced the ER stress marker IRE-1, but not ATF6 or PERK. IRE-1 activation promoted thioredoxin-interacting protein (TXNIP) translocation to mitochondria [202]. TXNIP was previously shown to have a different subcellular localization, but its translocation to mitochondria inactivates thioredoxins/trx proteins, which act as antioxidants by facilitating the reduction of other proteins by cysteine thiol-disulfide exchange, and increases ROS production [203, 204]. Increased formation of mitochondrial ROS induces NLRP3 translocation to the OMM and presumably to MAM, where caspase-2 translocation to the OMM is promoted. Here, caspase-2 cleaves and thereby activates BH3 interacting-domain death agonist (BID), which facilitates BAX/BAK-mediated mitochondrial DAMP release (Figure 2) [202]. However, it is not clear that activation of IRE-1 triggered by RB51 infection or treatment with LPS requires MAM formation. IRE-1 protein has previously been detected in MAM, where it was found to act as a ROS sensor. Exposure to high concentrations of ROS activates and stabilizes IRE-1 through interaction with the chaperone protein sigma-1 receptor (Sig-1R) on MAM. Activation of IRE-1 promotes XBP-1 activation as a protective response against ER stress [205]. However, the importance of MAM for IRE-1-mediated inflammasome activation requires further assessment.

**Figure 3:**
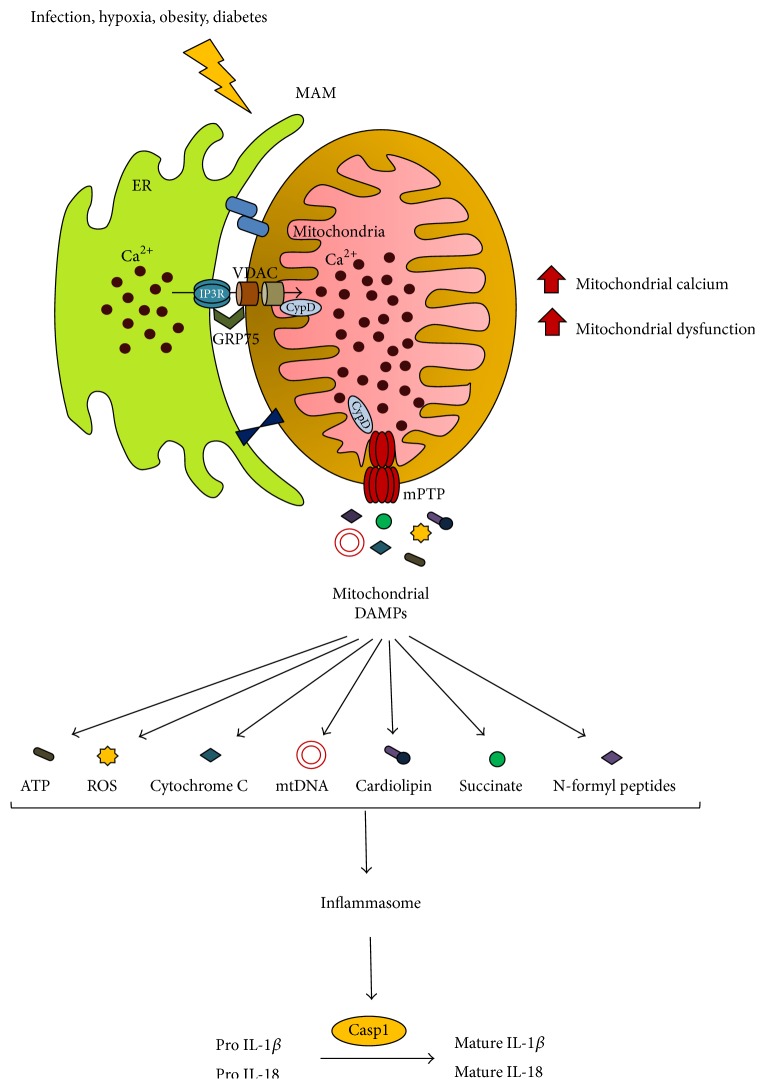
A hypothetical model of mitochondrial DAMP induction through mitochondria-associated ER membrane (MAM). Cellular metabolic dysfunction mediated by infection, hypoxia, obesity, and diabetes triggers abnormal association of endoplasmic reticulum (ER) and mitochondria. This increase in MAM formation induces mitochondrial calcium overload and exacerbates mitochondrial ROS generation, leading to mitochondrial permeability transition pore (mPTP) opening. The mitochondrial matrix protein CypD mediates mitochondrial calcium accumulation by physically interacting with the IP3R, GRP75, and VDAC complex at the MAM interface and modulates mPTP pore opening directly. mPTP opening facilitates release of mitochondrial components such as ATP, ROS, cytochrome C, mtDNA, cardiolipin, succinate, and N-formyl peptides into the cytosol, where they serve as mtDAMPS to activate the inflammasome. Subsequently, inflammasomes activate caspase-1 (Casp1) to induce cleavage and maturation of the proinflammatory cytokines IL-1*β* and IL-18. CypD: cyclophilin D; IP3R: inositol 1,4,5-trisphosphate receptor; GRP75: 75 kDa glucose-regulated protein; VDAC: voltage-dependent anion channel; mtDNA: mitochondrial DNA; IL-1*β*: interleukin-1 beta; IL-18: interleukin-18; ATP: adenosine triphosphate; ROS: reactive oxygen species; mtDAMPs: mitochondrial damage-associated molecular patterns.

The mitochondrial matrix protein cyclophilin D (CypD), which modulates opening of mPTP, was found to reside in MAM and to interact with the VDAC1, GRP75, and IP3R1 complex to regulate calcium transfer from the ER to mitochondria [13, 206]. During cardiac IR, CypD plays a dual role, inducing mitochondrial calcium accumulation and also promoting mPTP pore opening to induce apoptosis [13]. mPTP opening is a major mechanism responsible for the release of mitochondrial DAMPs into the cytosol and therefore inflammasome activation [207]. Stimulation of mouse macrophages with LPS enhanced mitochondrial damage and induced inflammatory responses, whereas, in macrophages lacking CypD, the LPS-stimulated inflammatory response was suppressed [208]. These findings indicate the direct involvement of MAM in the generation of DAMPs and inflammasome activation in metabolic diseases. Based on this current understanding, a hypothetical model of the effects of mitochondrial DAMPs through MAM is presented in Figure 3.

## 8. Association of MAM with Metabolic and Inflammatory Diseases

Metainflammation is a low-grade type of inflammation which is triggered by metabolic dysfunction [209]. Because of the numerous environmental, genetic, and psychosocial factors that promote it, metainflammation is an increasing threat to human health. Many reports have shown a link between inflammation and metabolic diseases such as atherosclerosis, diabetes, cancer, and AD [1, 210, 211]. Many defects in cellular pathways and cellular organelles have been implicated in the pathogenesis of these diseases. One of the critical common mechanisms is dysfunction of the ER and mitochondria, which are hubs for many signaling pathways. Cross-communication between these two organelles through formation of MAM has also been specifically implicated in many diseases [212]. The following table (Table 1) lists some of the diseases that have been associated with MAM and MAM-resident proteins.

## 9. Conclusion

MAM is enriched with master regulator proteins that influence multiple signaling pathways to determine cellular fate. Numerous studies have revealed its association with many pathological conditions ranging from pathogenic infections to diabetes and cancer. All these metabolic diseases are mediated through low-grade inflammation due to harmful environmental factors, especially overconsumption of an unhealthy diet. ER stress and mitochondrial stress are the first signs of cellular stress, and prolonged stress in these organelles leads to deterioration in their normal function, which can lead to the development of complex metabolic diseases. MAM provides a platform for cross-talk between the ER and mitochondria, allowing rapid exchange of biological molecules to maintain cellular health. However, abnormal exchange of these cellular metabolites, such as lipids and calcium, due to excess nutrient intake, hampers mitochondrial health and promotes increased production of ROS and release of mitochondrial DAMPs into the cytosol. These molecules are recognized by inflammasome components, triggering increases in proinflammatory cytokine secretion, followed by activation and sensitization of immune cells to initiate inflammatory responses.

In this review, we have elaborated the pivotal role of MAM in the instigation of metainflammation and the development of complex metabolic diseases. Frequent reevaluation and review of independent findings are needed to assist with the development of future therapeutics targeting these mechanisms. Overall, MAM has great potential to provide future therapeutic targets for a wide range of metabolic and inflammatory diseases.

## Figures and Tables

**Table 1:** MAM-resident proteins linked with metabolic and inflammatory diseases.

Proteins	MAM formation	Cell/tissue	Diseases	References
IP3R1, PACS2	Promote	Liver	Insulin resistance	[6]
CypD	Promote	Liver	Insulin resistance	[23]
CypD	Promote	Cardiac muscle	Ischemia-reperfusion injury	[13]
GSK-3*β*	Promote	Cardiac muscle	Ischemia-reperfusion injury	[24]
Mfn2	Promote	Human airway smooth muscle cells	Asthma	[25]
PML	Promote	Mouse embryonic fibroblast cells (MEFs)	Cancer	[20]
p53	Promote	Various cancer cells	Cancer	[26]
p66Shc	Promote	MAFs (mouse adult fibroblasts)	Cancer	[27]
PTEN	Promote	HEK-293, MEFs	Cancer	[28]
FATE1	Inhibit	Cancer cells	Cancer	[29]
TMX1	Promote	Cancer cells, MEFs	Cancer	[30]
FUS	Promote	Neuronal cells	ALS	[31]
Presenilins	Promote	Brain	Alzheimer's disease	[10, 32]
Parkin	Promote	Brain	Parkinson's disease	[33]
*α*-Synuclein	Promote	Brain	Parkinson's disease	[34]
Nogo A	Inhibit	Cardiomyocyte	Ischemia-reperfusion injury	[35]
Nogo B	Inhibit	PASMCs, CASMCs, RASMCs	Pulmonary hypertension	[36]
RyR, VDAC	Promote	Cardiac muscle	Aging	[37]

IP3R1: inositol 1,4,5-trisphosphate receptor 1; PACS2: phosphofurin acidic cluster sorting protein 2; CypD: cyclophilin D; GSK-3*β*: glycogen synthase kinase 3 beta; Mfn2: mitofusin 2; PML: promyelocytic leukemia protein; p53: tumor protein p53; p66sh: 66 kDa isoform of the growth factor adapter Shc; PTEN: phosphatase and tensin homolog; FATE: fetal and adult testis-expressed transcript protein; TMX1: thioredoxin related transmembrane protein 1; FUS: fused in sarcoma; Nogo A and B: reticulon-4/neurite outgrowth inhibitor; RyR: ryanodine receptors; VDAC: voltage-dependent anion channel; ALS: amyotrophic lateral sclerosis; PASMCs: porcine aortic smooth muscle cell; CASMCs: chicken aortic smooth muscle cells; RASMCs: rat aortic smooth muscle cell.
